# Treatment of Teeth with Dead Pulps

**Published:** 1873-10

**Authors:** 


*Treatment of Teeth with Dead Pulps.*—Dr. I. N. Krouse, of Chicago, "thinks it much better to treat this class of teeth too often than not often enough. If they were always treated properly before filling, we should not have so many alveolar abscesses. Uses creasote a number of times, getting to the end of each root if possible, so that every particle of decomposing matter, and the gases arising therefrom, are destroyed. Dr. Glidden's drills are superior to any we have in the depots, which may account, in part, for his success in filling dead and abscessed teeth without previous treatment. Yet the speaker could not agree with this practice. As before said, we must have all decomposition stopped and the root in a healthy condition, before filling, and in case of alveolar abscess, the sack and growth at the end of root must be destroyed. Knows of nothing better to accomplish this than creasote. His plan of getting this through the fistulæ is with India rubber. Makes a piston of it by cutting it something of the shape of the carious cavity, then fills the root with creasote and pumps with the rubber piston, making quick and rapid motions, using some blunt instrument that will not pierce the rubber. Often succeeds in getting the creasote through without enlarging the pulp-canal, but when we cannot succeed without drilling and enlarging the canal, this should be done. He makes a drill for these special cases, filing it very small, so that it is limber a considerable way, then makes a good spring temper, and it works admirably. He much prefers, however, not to drill through and enlarge the opening at apex, and never does it unless he cannot succeed in forcing the creasote through the fistulæ. Would then close the tooth with temporary filling, if it was likely to be stubborn about getting well, before filling permanently.

Dissolved Hill's stopping is very good to fill certain cases, as where the root is crooked and when impossible to fill with gold with any certainty ; but in most cases fills with gold after plan suggested by Dr. M. S. Dean—with No. 20 or 30 foil. Thinks in this way roots can be filled, in a majority of cases, better than with any other substance. Next to this, perhaps, comes gutta percha, or Hill's stopping dissolved in chloroform. It has the advantage of not requiring so much skill or care in its manipulation. The treatment of the case spoken of by Dr. DeCrow, could not be very definitely determined here without an extensive description. We must have all the circumstantial as well as the direct evidence. The former habits of the patient should be known. Some constitutional difficulty, perhaps syphilis, is at the bottom of the trouble. It has been recommended to extract and replace the tooth. If every other known remedy has failed, and there is no prospect of nature setting up a healthy action, then it may be allowable to extract the tooth. The practice of extracting and replacing teeth should be the very last resort and only practiced as such. Within the last few years we have been hearing considerable about this plan of treating alveolar abscesses. Had but little faith in it himself. Cares not what is done with the tooth after it is extracted, whether it is scraped, or the ends of roots cut off, soaked in carbolic acid, or kept in tepid water, till the periosteum is restored or made healthy. To make it a general practice is not rational, and does not belong to the nineteenth century."

